# Personal selling in health and medicine: using sales agents to engage audiences

**DOI:** 10.1186/s12913-020-05600-z

**Published:** 2020-09-15

**Authors:** James K. Elrod, John L. Fortenberry

**Affiliations:** 1 Willis-Knighton Health System, 2600 Greenwood Road, Shreveport, LA 71103 USA; 2 LSU Shreveport, 1 University Place, Shreveport, LA 71115 USA

**Keywords:** Personal selling, Marketing communications, Promotion, Hospitals, Healthcare

## Abstract

**Background:**

Personal selling—the use of sales agents to personally deliver messages to target audiences—is often not the first conveyance pathway that comes to mind when thinking about marketing communications in the health services industry. This is not surprising given that sales force roles are not as public and prominent as other promotional avenues, such as advertising and public relations. Further, the titles held by those in sales-oriented roles in the health services industry are usually more discreet, carrying designations such as community liaison, business development officer, and the like. Regardless of title, sales roles involve personally interacting with desired audiences to compel some sort of action, adding a vital form of communication that bolsters engagement opportunities.

**Discussion:**

Personal selling plays a critical role in the promotion of health services organizations. Perhaps most obviously, it is distinguished from its counterparts in the marketing communications mix by its use of people to deliver messages to desired audiences. Associated titles, duties, and expectations vary widely between and among those healthcare entities which make use of personal selling, as there is no pat formula for deployment within health services environments. To shed light on personal selling, this article presents an associated overview through the lens of Willis-Knighton Health System, sharing practical insights and experiences which can assist peer healthcare establishments in understanding, shaping, and honing sales roles within their own facilities.

**Conclusions:**

Taking advantage of the utility afforded by direct, personal interactions with audiences, personal selling provides a helpful communications resource that better enables healthcare providers to connect proficiently with target markets. It supplements other forms of marketing communication, operating synergistically to help healthcare institutions achieve their conveyance goals. Prudent deployment of this unique marketing communications method affords health and medical institutions with a capable conveyance asset that can provide great assistance in achieving communicative ambitions.

## Background

Meaningful audience engagements are essential for health and medical organizations, compelling patronage which fosters institutional growth and prosperity [1–5]. Success on this front yields the obvious benefits of viability and vitality for given health services organizations, but it also delivers benefits that extend beyond the walls of these institutions, encouraging audiences to attend to their healthcare wants and needs, ultimately affording a healthier populace [2, 6, 7]. The mutual benefits gained by communicating well should remain at the forefront of thought for healthcare providers, prompting them to pursue marketing communications excellence as voraciously as they do healthcare delivery excellence.

When one thinks about marketing communications in the healthcare industry, very often images of billboard advertisements, television commercials, direct mail parcels, and similar conveyances come to mind. This perspective is not surprising given the prominence and persistence of these promotional avenues, especially when driven intensively by health and medical establishments in an effort to reach target audiences. But less obvious communication pathways also exist, with one of these being personal selling which involves the use of sales agents to personally deliver messages to target audiences [2, 8]. The notion of the sales representative often evokes images of occupations far removed from the health services environment, but sales roles indeed exist in health services establishments and they are very important [2, 5, 9–11].

Pat formulas for effecting personal selling in health services organizations do not exist, as institutions and their deployment needs vary, leading to variations in titles, roles, and missions. Still, it often is helpful to observe the personal selling efforts of peer healthcare facilities when possible, as doing so can provide informative details, permitting opportunities for reflection and refinement. Gaining such insights, however, is somewhat rare, given the competitive nature of the health services industry, but some healthcare establishments are willing to share accounts of their personal selling insights and experiences, with this particular article forwarding those of Willis-Knighton Health System, bolstering the experiential profiles available in the literature and supplying operational guidance for health and medical providers.

## Definition and overview

Personal selling is a key communicative component of the broad discipline of marketing, formally defined as “a management process that involves the assessment of customer wants and needs, and the performance of all activities associated with the development, pricing, provision, and promotion of product solutions that satisfy those wants and needs” [2], p. 288. Promotion, as evidenced in this definition, is a core feature of marketing, earning inclusion as one of the Ps in the classic expression known as the *four Ps of marketing* (i.e., Product, Price, Place, Promotion). The promotion aspect of marketing essentially entails any and all elements associated with engaging audiences, with the core pathways for engagement being depicted in a descriptive model known as the marketing communications (or promotions) mix [1, 2].

The marketing communications mix, as traditionally depicted, contains five principal avenues of communication; namely, advertising (i.e., the paid use of mass media to deliver messages), personal selling (i.e., the use of sales agents to personally deliver messages), sales promotion (i.e., the use of incentives, such as contests and free giveaways, to encourage patronage), public relations (i.e., the use of publicity and other unpaid promotional methods to deliver messages), and direct marketing (i.e., the delivery of messages via mail, the Internet, and similar routes directly to consumers) [2, 8]. Healthcare providers examine each of these communicative avenues, selecting one or more believed to be most capable of reaching target audiences, with the ultimate goal being to encourage patronage or compel some other desired action [2, 12].

Personal selling is used in the health services industry in situations where communicative challenges require establishing a personal, interactive dialogue with sought audiences [1, 2, 8–11]. Personal selling through its communicative vehicle, the sales agent, is capable of sending messages, receiving responses from recipients, reacting to those responses, and continuing an established dialogue with one or more parties involved in the particular engagement [2, 13, 14]. This, of course, gives personal selling a dramatic capability over advertising, perhaps the most utilized component of health services marketing communication, as message dissemination through mass media channels is unidirectional and nonpersonal (i.e., not delivered in-person). Even with direct marketing, which notably includes telemarketing, social media, and other interactive avenues [2, 13], personal selling has an advantage in that it incorporates face-to-face, real world, in-person communications, which, for some missions, are imperative [8, 10, 13, 14].

Personal selling is deployed in many different ways across the health services industry, with implementation characteristics being dependent on the missions called for by given healthcare providers. Roles are often titled discreetly to reduce the overly commercial tone of descriptors such as sales representative or sales agent, opting instead for titles that are softer, such as community liaison, business development officer, and so on. Regardless of title, such roles involve personally interacting with desired audiences to compel some sort of action, adding a vital form of communication that bolsters opportunities to engage audiences [2, 8–10, 13, 14].

Nursing homes, for example, are dependent on referrals from families, hospitals, social services agencies, and other establishments in their given markets. In pursuing these referrals, personal selling often is used, with representatives calling on sources, seeking to encourage associated patronage. Without personal, direct contact, referrals would dwindle and the livelihoods of given nursing homes would be in jeopardy. Personal selling agents working on behalf of more diversified healthcare entities, like major hospitals, often have broader outreach-related roles. They, for example, might be called upon to engage referral sources, attend community events as representatives of their given healthcare establishments, provide informative presentations about the health services of their institutions at civic clubs and other venues, meet with politicians and other dignitaries to discuss community health matters, and so on. Those engaged in personal selling play critical roles which positively contribute to the overall communications strategies of healthcare providers [2, 8–10].

## Institutional background, deployment history, and context within marketing communications

Established in 1924, Willis-Knighton Health System possesses a rich history of successfully communicating with audiences. Communications excellence, in fact, has been and continues to be viewed as a strategic priority, compelling extensive use, experimentation, and innovation. Based in Shreveport, Louisiana and situated in the heart of an area known as the Ark-La-Tex where the states of Arkansas, Louisiana, and Texas converge, Willis-Knighton Health System holds market leadership in its served region where it delivers comprehensive health and wellness services through multiple hospitals, numerous general and specialty medical clinics, an all-inclusive retirement community, and more. The achievement of market leadership can be credited, in part, to communications prowess, permitting the institution to effectively engage customer groups, yielding significant interest and attention, ultimately leading to all-important patronage and customer loyalty.

Today, Willis-Knighton Health System leverages the power of the full marketing communications mix, deploying all of its components, including personal selling, with this particular element having an intriguing institutional history. Customer care and attention roles, community outreach initiatives, business development directives, and similar sales-oriented pursuits have always been a requirement of the establishment. Prior to the 1970s, it was not uncommon for key executives to hold simultaneous duties as sales agents of the establishment. During this era, the institution played a relatively small role in the delivery of health services in the greater Shreveport-Bossier City marketplace which was dominated by larger, better resourced competitors. Resource scarcity demanded economization at every turn, which often translated into employees holding a range of responsibilities well beyond what would constitute usual and customary duties associated with their official titles [15]. As the institution developed and resources increased, however, specialization became possible, permitting the hiring of personnel dedicated to sales roles.

Formalization of personal selling roles became particularly robust in the 1970s when Willis-Knighton Health System embarked on an aggressive growth campaign, desiring an expanded geographic footprint. To achieve this bold vision, the institution pursued a range of growth-fueling innovations, including adoption of the hub-and-spoke model [16, 17], establishing centers of excellence [18], expanding physical space via the practice of adaptive reuse [19, 20], and more. Knowing that these new initiatives would count for very little without attracting increasing numbers of patients, Willis-Knighton Health System carefully assessed its communications efforts, seeking to shore up capabilities to ensure that it could meet its growth ambitions. Personal selling roles, notably including those involving community outreach, business development, and related missions were slated, with these continuing to evolve as the institution’s fortunes increased.

Today, personal selling remains a valuable component of Willis-Knighton Health System’s marketing communications strategy. It primarily is used as a strategic communications enhancement, complementing other components of the marketing communications mix, with it being deployed for missions that are difficult or impossible for nonpersonal methods of communication (e.g., advertising) to fulfill. Willis-Knighton Health System’s business development officers, for example, are charged with engaging community influencers. These notably include leaders of churches, educational institutions, military establishments, health insurance companies, and other organizations with populations which might benefit from Willis-Knighton Health System’s services. Further, as designated community liaisons, the institution’s business development officers are highly involved in community organizations, such as Rotary, Kiwanis, the Chamber of Commerce, and related establishments. Through these interactions, agents are able to learn about the needs of institutions and individuals in Willis-Knighton Health System’s served markets, share opportunities for the provision of assistance, nurture existing patronage and build new patronage, and foster general goodwill.

Business development officers are highly knowledgeable of Willis-Knighton Health System’s history, service array, community initiatives, and related programs, permitting them to speak with authority to others as they make rounds in the community, building valuable connections. Sometimes human delivery and associated interactions between parties are required to effectively disseminate communications, address inquiries, and secure desired actions, with personal selling being the operative avenue for such missions. The highly-distinguishing human touch characteristic afforded by personal selling has compelled Willis-Knighton Health System to make use of this particular communicative pathway as a means of pursuing challenging communications goals and objectives.

## Strengths

Willis-Knighton Health System’s personal selling experiences suggest a number of strengths, with these compelling its historic and continued use. Chief attributes of personal selling prompting its use are described as follows.

### Engagement flexibility

Personal selling permits two-way, interactive dialogues between sales agents and their audiences, giving a vital human touch to associated engagement opportunities [2, 13, 14, 21, 22]. This stands in sharp contrast to the vast majority of marketing communications which are unidirectional, conveying information but not permitting receipt of responses via the disseminating channel, and nonpersonal, relying on delivery methods which do not involve in-person interactions [2, 8, 13, 23, 24]. Advertisements encouraging patients to visit particular medical centers, direct mail pieces compelling patient patronage, and press releases carried in newspapers which announce new medical services at hospitals serve vital purposes, but without the ability to receive replies and respond in-person and in real-time, communications potential remains limited. While those exposed to various nonpersonal forms of promotion certainly can telephone given healthcare facilities for more information or pursue other pathways to address lingering questions, the ability to inquire immediately with a sales agent represents perhaps the most ideal form of engagement possible.

### Potential to build relationships

The in-person, interactive aspect of personal selling gives this particular communications pathway a characteristically human nature, permitting the establishment of productive relationships between sales personnel and target audiences [2, 13, 14, 21, 22]. Just as personal relationships are advanced through direct, human interactions, so too are professional relationships, including those between the agents of healthcare establishments and their clients, whether those clients happen to be patients, peer institutions, or other publics. Personal interactions between sales agents and their target audiences over time engender trust which sets the stage for mutually beneficial exchange to occur.

### Potential to bolster market intelligence

Courtesy of their exposure to audiences, those engaged in personal selling endeavors on behalf of healthcare institutions are ideally situated to collect market intelligence [2, 13, 14, 21, 22]. Through interactions with current and prospective patients, peer healthcare institutions, community organizations, politicians, and other publics, as well as exposure to the sights and scenes of marketplaces, individuals holding sales-oriented roles are positioned to acquire vast amounts of information. These insights often shed light on sources of satisfaction and dissatisfaction, opportunities for improvement, desired healthcare services, emerging competitive threats, and related facets that can help healthcare institutions elevate their service competencies, fend off competitors, and better address their constituents. Such perspectives also have the potential to shed light on findings from existing marketing research efforts, such as patient satisfaction surveys, focus groups, customer advisory panels, and other investigative avenues, bolstering institutional intelligence, permitting improved strategies and tactics.

## Limitations

The strengths motivating selection of personal selling as an engagement pathway are moderated by a series of limitations which must be factored into associated evaluations. Notable limitations are described as follows.

### High cost

Personal selling is a costly endeavor. People, serving effectively as the medium of communication, are expensive, requiring the usual resources of salaries, benefits, supervision, training, and so on [2, 13, 14, 21, 22]. Assuming the given individuals are skilled and deployed in a proper manner, however, health and medical institutions should expect an acceptable return on investment. Further, very often those involved in personal selling roles on behalf of healthcare institutions hold additional duties and responsibilities, widening opportunities to recoup and capitalize on expenses associated with fielding sales forces. Even if individuals are completely dedicated to sales-oriented roles, multitasking often is possible, affording opportunities for sales agents to engage in ancillary activities related to their given duties. Other forms of marketing communication by contrast offer limited dimensionality as quite typically, they can only serve as conveyance mechanisms, with associated costs resting only on that particular role.

### Limited reach

A defined weakness of personal selling is that of limited reach. The number of clients that an individual sales agent can contact and address quite obviously is limited, simply due to the finite capacity of the given representative [2, 13, 14, 21, 22]. By contrast, a single promotion, such as a billboard advertisement, can reach thousands quickly and easily, depending on placement characteristics [2, 8, 23, 24]. Reach limitations, however, should not be viewed in an entirely negative fashion. Sales roles ideally should be used to supplement other forms of marketing communication, with each being deployed in a manner to play on its given strengths. Those forms capable of delivering conveyances to large audiences ultimately support sales agents in the field who deliver communications more strategically toward defined targets that require additional communicative support. It is simply a matter of using the components of the marketing communications mix in an appropriate manner, affording opportunities to derive the most from each pathway and collectively achieve communications goals.

### Potential for inconsistency

Unlike other forms of marketing communication where promotional materials can be reviewed and approved before deployment, offering reasonable assurances that the messages will be transmitted exactly as intended, personal selling offers no such guarantees [2, 13, 14, 21, 22]. This, of course, is due to the inevitable differences between and among individuals serving in sales roles. People vary in their competencies, dedication, perseverance, and commitment, among many other qualities, with this ultimately influencing personal selling outcomes. The best method for reducing the potential for inconsistency among those in sales-oriented roles is to recruit effectively to ensure that candidates possess excellent qualities for engaging audiences. Attention also must be directed toward the provision of excellent training and proper supervision, ensuring that those in sales roles possess the skills and guidance required for success. Of course, anyone can face hardships, personally or professionally, impacting their performance, so regardless of steps taken to ensure consistency, the potential for variation is ever present. The human factor in personal selling in this particular case represents a weakness, but this very factor also delivers the avenue’s chief strengths.

## Operational reflections

For administering any component of the marketing communications mix, Willis-Knighton Health System advises establishing a baseline foundation of resources, including (1) top leadership support and commitment, (2) financial resources sufficient for funding communications activities, (3) competent personnel charged with effecting given initiatives, and (4) formal processes permitting effective planning, implementation, and evaluation of initiatives. Adequate resources set the stage for productive audience engagement endeavors, minimizing chances of resource-depleting and reputation-damaging mistakes which, in the realm of marketing communications, often are very public, given the open circulation of such conveyances. These resources also ensure competencies in using given marketing communications mix components, with proper deployment being essential for realizing desired outcomes.

As for personal selling, specifically, beyond the advisories presented elsewhere in this article, operationally Willis-Knighton Health System recommends that healthcare providers reflect on their given workforces, identifying all individuals involved in audience engagement pursuits, ensuring that their contributions and insights are factored into the greater marketing communications plans of given entities. Failures to do so represent missed opportunities to make use of valuable marketplace intelligence acquired by sales agents, courtesy of immersion in their respective communities. Exclusion of such also diminishes abilities to integrate personal selling with other components of the marketing communications mix. Imagine, for example, the profound loss of intelligence that would occur if Willis-Knighton Health System neglected to tap into the wealth of insights collected by its business development officers. Indeed, marketing communications campaigns must link each and every communicative element together to present a consistent story to audiences, something which affords integrated marketing communications. Since sales agents sometimes are overlooked and omitted from greater marketing communications planning activities, healthcare establishments would do well to take positive steps to ensure their inclusion, permitting these individuals to make the highest contributions possible to advance the conveyance initiatives of their employers.

## Conclusions

Taking advantage of the utility afforded by direct, personal interactions with audiences, personal selling provides a helpful communications resource that better enables healthcare providers to connect proficiently with target markets. It supplements other forms of marketing communication, operating synergistically to help healthcare institutions achieve their conveyance goals. Care must be taken to deploy personal selling properly, capitalizing on its strengths while minimizing or avoiding its limitations. Healthcare institutions also must ensure that they accurately identify all individuals under their employ who are engaged in personal selling initiatives, seeking their full engagement in marketing communications planning. Prudent deployment of this unique marketing communications method affords health and medical institutions with a capable conveyance asset that can provide great assistance in achieving communicative ambitions.

## Data Availability

Not applicable.
